# Early Spatial Memory Impairment in a Double Transgenic Model of Alzheimer’s Disease TgF-344 AD

**DOI:** 10.3390/brainsci11101300

**Published:** 2021-09-30

**Authors:** Stephanie L. Proskauer Pena, Konstantinos Mallouppas, Andre M. G. Oliveira, Frantisek Zitricky, Athira Nataraj, Karel Jezek

**Affiliations:** Biomedical Center, Faculty of Medicine in Pilsen, Charles University, 30605 Pilsen, Czech Republic; constantinosmal@hotmail.com (K.M.); andreoliveiragm@hotmail.com (A.M.G.O.); Frantisek.Zitricky@lfp.cuni.cz (F.Z.); athira.nataraj@lfp.cuni.cz (A.N.)

**Keywords:** Alzheimer’s disease, spatial memory, navigation, TgF-344 AD

## Abstract

Before the course of Alzheimer’s disease fully manifests itself and largely impairs a patient’s cognitive abilities, its progression has already lasted for a considerable time without being noticed. In this project, we mapped the development of spatial orientation impairment in an active place avoidance task—a highly sensitive test for mild hippocampal damage. We tested vision, anxiety and spatial orientation performance at four age levels of 4, 6, 9, and 12 months across male and female TgF-344 AD rats carrying human genes for presenilin-1 and amyloid precursor protein. We found a progressive deterioration of spatial navigation in transgenic animals, beginning already at the age of 4 months, that fully developed at 6 months of age across both male and female groups, compared to their age-matched controls. In addition, we described the gradual vision impairment that was accentuated in females at the age of 12 months. These results indicate a rather early onset of cognitive impairment in the TgF-344 AD Alzheimer’s disease model, starting earlier than shown to date, and preceding the reported development of amyloid plaques.

## 1. Introduction

Alzheimer’s disease (AD) is a human neuropathology characterised by a progressive loss of cognitive functions, severe neurological deterioration and subsequently leading to death [1,2]. The anatomical signs associated with AD are mainly represented by a formation of deposits of amyloid-β (Aβ), neurofibrillary tangles (NFT) of hyper-phosphorylated Tau, and by neuroinflammation associated with progressive neuronal loss [2,3]. In its natural form, AD is exclusively expressed in humans. Whereas Aβ plaques similar to those observed in human AD patients were described in other mammals, their combined presence with NFT is almost absent in non-human species [4,5].

Advancements in genetic engineering enabled the design of numerous genetic models of AD, mainly in mice. A majority of them hold a mutated gene for amyloid-β precursor protein (APP), and/or presenilin 1 (PS1) gene, as their mutations were identified in the familiar form of AD [6,7]. Recent efforts to provide more complex models of AD has led to a development of transgenic AD rat constructs [8,9] reflecting the fact that rats are about 5 million years closer in evolution to humans than mice and that their brains carry an approximately three -times higher number of neurons [10].

The rat model TgF-344 AD is built on a platform of Fisher-344 phenotype, by inserting the human mutated genes for amyloid precursor protein APP (“Swedish” mutation, APPsw) and ∆ exon 9 presenilin-1 PS1 ∆ E9AβPP [11]. The subjects show a formation of amyloid beta deposits, tangles of hyper-phosphorylated Tau, neurodegeneration and cognitive impairment [11,12,13,14]. 

Both in the clinical and experimental research, considerable effort is focused on detecting the early onset signs of the disease. In this respect, deterioration of spatial cognition has been identified as one of the earliest behavioural AD manifestations [15,16,17,18]. Indeed, the main neuronal components of the brain spatial navigational system overlap with the hippocampo-entorhinal networks [19,20,21] that have been found structurally altered early in the course of disease development [22,23]. 

The development of spatial cognition decline in TgF-344 AD has been the subject of several studies since its introduction in 2013. While studies differ on the reported onset of spatial memory impairment, they also contrast with recent findings on the early response in network oscillatory properties in TgF-344 AD [24]. The earliest reported age level with clearly expressed spatial memory impairment was detected in 10- to 11-month-old TgF-344 AD animals [25]. Stoiljkovic et al. (2019) and Smith and McMahon (2018) [26], on the other hand, showed already in 6-month-old transgenic rats of the same strain, an affected synaptic plasticity and broadly altered oscillatory activity in theta and gamma bands, including impaired hippocampo-neocortical network dynamics. Since the theta/gamma rhythmicity is essential for spatial information processing in the hippocampo-entorhinal circuitry [27,28,29], we hypothesised that the changes reported in Stoiljkovic et al. (2019) should be reflected in an impairment of the ability to navigate, especially in the navigation strategies that critically depend on the integrity of the hippocampo-entorhinal loop. Because the ability to navigate in a space is controlled by multiple information processing pathways, its specific hippocampal contribution in early stages might be masked in certain behavioural tasks by using alternative strategies. In other words, testing navigation under selective involvement of its allothetic (hippocampus-dependent) component with high information processing demand, might more accurately reveal its impairment onset. To detect even subtle spatial memory impairment, we employed the Active Allothetic Place Avoidance task (AAPA, [30]), a task that selectively tests the distal cues-based allothetic component of spatial navigation while requiring the use of idiothesis to be suppressed, as well as the use of local intra-maze cues (such as odours, etc., used to mark the relevant spatial locations). The necessity to split the idiothetic and allothetic navigation frames was shown to detect a mild hippocampal damage that remained unnoticed by more conventional navigation tests in animals [30,31,32], as well as in humans [16,17]. 

The goal of our study was to identify the onset of allothetic spatial navigation impairment in TgF-344 AD by testing the age levels at 4–5, 6–7, 9–10 and 12 months. Because the course and impact of AD in humans shows a sex-specific difference, we hypothesised that the dynamics of impairment in navigation might differ between females and males in TgF-344 AD too. Therefore, the sexes were tested independently wherever possible. 

The results indicate that spatial navigation impairment in TgF-344 AD rats was reliably detectable already at 6–7 months, with signs of impairment even at 4–5 months of age, earlier than reported previously by using the Morris Water Maze [25] and the Barnes Maze procedure [11], respectively. While the spatial memory defect was similar across males and females, we found that the sexes were differently impaired in sight deterioration, as the females expressed stronger impairment in the oldest (12 months) age group. 

## 2. Materials and Methods

### 2.1. Subjects

All protocols followed in this study were approved by the Ethical Committee of the Ministry of Education, Youth and Sports of the Czech Republic (approval no. MSMT-12048/2019-14) according to the Guide for the Care and Use of Laboratory Animals (Protection of Animals from Cruelty Law Act No. 246/92, Czech Republic). One hundred and eighty-seven rat subjects of the TgF-344 AD strain (*n* = 95) and their F-344 controls (*n* = 92), obtained from the local breeding colony at the Faculty of Medicine in Pilsen, Charles University, were used in the study. The animals were tested at independent age levels of 4–5, 6–7, 9–10 and 12 months. For each of the 6–7, 9–10 and 12-month cohorts, we built 4 independent groups based on their sex (males (M) × females (F)) and phenotype (transgenic (EXP) × controls (CTRL)). The 4- to 5-month-old animals were only males (EXP vs. CTRL). The individual group sizes are listed in Table S1. All rats were handled approximately 5 min per day for the period of 21 days before the testing started. A tail sample (2 mm) was collected across all subjects at the age of 8–14 weeks, extracted by using QIAGEN DNeasy Blood and Tissue Kit (QIAGEN, Valencia, CA, USA). The presence of transgenes was identified by PCR, using APP (Forward- 5′-CCG AGA TCT CTG AAG TGA AGA TGG ATG-3′) and PS1 (Forward- 5′-CAG GTG GTG GAG CAA GAT G-3′) primers.

### 2.2. Behavioural Testing

All animals were tested in a sequence of three tasks: the visible platform version of the Morris Water Maze (MWM) for vision assessment (days 1 and 2), Active Allothetic Place Avoidance task (AAPA) to test the spatial memory (days 3–10), and Elevated Plus Maze (EPM, day 11) to estimate the level of anxiety. 

#### 2.2.1. Vision Test

The visible platform version of the Morris Water Maze (MWM) consisted of a white plastic circular tank (180 cm in diameter, 60 cm high walls), filled with water (20 °C, 40 cm deep). A metallic visible platform (15 cm in diameter) was placed 0.5 cm above the water surface in the centre of the south-west quadrant of the pool and was marked by a 25-cm-high bar with black and white horizontal stripes. 

Rats were released facing the wall from the four starting points (E, W, S, N), and the time to locate the goal was manually measured. A typical trial finished when the rat found the labelled platform. If the rat failed to find the goal within 60 s, the trial was stopped, and the rat was placed on the platform by the experimenter. After reaching the goal in either way, it was left there for another 30 s and then returned into a waiting cage. Four trials with an inter-trial interval of 3 min were performed on each of the two days of testing. Animals that did not locate the marked platform within 60 s in more than 2 trials on the second day, were excluded from the study. 

#### 2.2.2. Active Allothetic Place Avoidance (AAPA)

The AAPA apparatus consisted of a stainless-steel circular arena (82 cm in diameter, enclosed with a 30 cm high transparent Plexiglas wall) placed 1 m above the floor in a moderately lit room with numerous available orientation cues. 

One week before the training, all rats were implanted, under mild isoflurane anaesthesia, with a hypodermic needle placed in the interscapular region and protected on both ends to provide a conductive contact for a later footshock delivery. For the testing, the rat wore a small backpack with an infrared LED for tracking and the piercing on its back was connected to the apparatus through a small alligator clip and the overhead balanced cable.

In AAPA, the animals had to avoid an invisible shock area on a slowly rotating arena (1 revolution per minute) by distal landmark-based orientation. The radial sector (60 °) was defined as stable in respect to the distal room cues, so that the animal had to move constantly in order not to be brought into it by the arena rotation. Upon the zone entry for more than 0.5 s, a 50 Hz current (0.2–0.6 mA) was delivered for 0.6 s. The shock was repeated every second until the animal left the zone. 

The rotation of the arena, rats’ tracking, and the delivery of the footshock was controlled by the Interactive tracking system (BioSignal Group, Brooklyn, NY, USA).

The behavioural training consisted of two days of habituation and six days of testing. One 20-min session was carried out per day. At the beginning of each session, the rat was placed on the rotating arena (1 rpm), facing the wall in a place opposite to the shock sector. The rats were first habituated to the apparatus on a non-rotating (habituation 1) and rotating (habituation 2) arena, respectively, with the footshock disabled. Thereafter, six daily test sessions were performed on the rotating arena with the footshock delivered whenever the animal entered the punished sector. 

The actively travelled distance, number of entries, number of obtained footshocks, and maximum avoided time and path, respectively, were recorded. The collected data were analysed using commercial software (TrackAnalysis, BioSignal Group, Brooklyn, NY, USA).

We report five main parameters assessing the cognitive and non-cognitive aspects of the task performance. Spatial memory was judged by counting the number of entries into the punished zone (spatial errors), the number of footshocks received, and the maximal path (maximal path avoidance, MPA) and time (maximal time avoidance, MTA) each animal was able to avoid the punished sector within the given session. Total distance travelled on the arena surface was measured as a non-cognitive output reflecting the general motion of the animal.

#### 2.2.3. Elevated Plus Maze (EPM)

The Elevated Plus Maze was used to assess the level of anxiety across all age groups. The testing took place two days after finishing the AAPA task. The apparatus consisted of two open and two closed arms 70 cm long and 10 cm wide, crossing perpendicularly in the centre. The closed arms were surrounded by 30-cm-high opaque walls and the whole maze was elevated 50 cm above the room floor. The rodents were placed in the centre of the maze and allowed to move freely for 10 min while recorded. The path travelled and the proportion of time spent in the closed arms were measured. To be categorised as closed arm presence, the whole rat body including its head had to be detected inside the closed arm. 

### 2.3. Statistics

The comparisons were performed using two-way ANOVA with or without repeated measures, with factors of day, group (transgenic × wild) and sex, respectively. Whenever suitable, the Mann–Whitney U test and chi square test were performed.

## 3. Results

### 3.1. Visible Platform Water Maze Test

**Figure 1:**
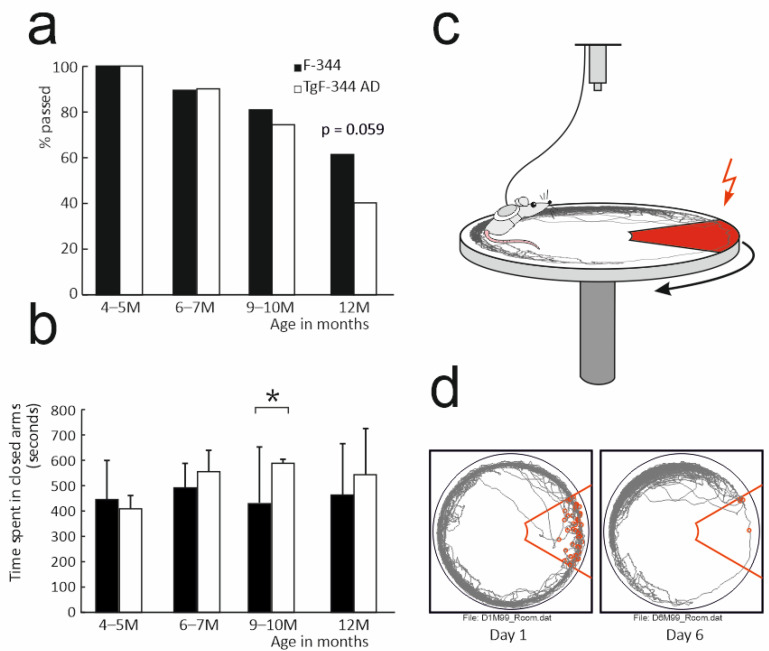
(**a**) Proportion of animals passing the visible platform Water Maze test (in percentage). (**b**) Time spent in the closed arms of the Elevated Plus Maze (median/interquartile range). The significant between-group comparison is marked with an asterisk. (**c**) Schematic drawing of the AAPA apparatus. The red area indicates the zone where the footshock was delivered upon entry (not marked in real situation). The arrow shows the direction of the arena rotation. (**d**) Examples of cumulative tracks (grey line) in the room coordinates frame from sessions on the first (left) and on the last day (right) of the training of one animal. The red lines show the contours of the zone with the footshock. The red circles indicate places where the footshocks were delivered.

In the visible platform Water Maze task, the proportions of animals that matched the criteria gradually decreased with the higher age (Figure 1a). While the first three age levels did not show significantly different fractions in passing the test between transgenic and control animals (X2 test, all *p* > 0.59), at the age of 12 months the percentage was smaller at the border of significance (X2 (1.83) = 3.56, *p* = 0.059) in transgenic subjects. When further splitting the 12-month-old animals based on sex, we found the female groups differed significantly (X2 (1.47) = 4.62, *p* = 0.032, Table S1, Figure S1a), while the proportions among males were indistinguishable (X2 (1.36) = 0.09, *p* = 0.765). The animals that passed the test were assigned into groups for subsequent testing. Their visual Water Maze test escape latencies across the second day were then comparable across all age- and sex-matched group pairs (all pairs *p* > 0.389, Figure S1b).

### 3.2. Elevated Plus Maze

In the Elevated Plus Maze test, we compared the cumulative time spent in the closed arms of the apparatus (Figure 1b). The two-way ANOVA revealed a significant factor of group (F (1.125) = 4.58, *p* = 0.034) and a non-significant factor of age (F (3.125) = 0.93, *p* = 0.431) and a group × age interaction (F (3.125) = 1.73, *p* = 0.164). The post-hoc tests showed the 9-month-old transgenic animals spent a significantly longer time in the closed parts of the maze than their age-matched controls (*p* = 0.001). The transgenic and control animals in the other age levels (4–5, 6–7 and 12 months) did not differ significantly (*p* > 0.05 in all cases).

### 3.3. Active Allothetic Place Avoidance Test

**Figure 2:**
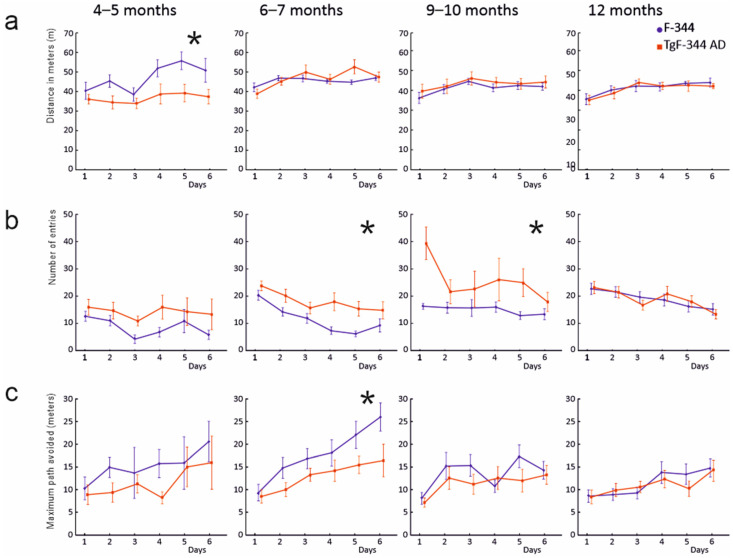
Spatial learning and memory in Active Allothetic Place Avoidance task across all age groups. Transgenic animals are marked in red; wild type animals in blue. Values are depicted as group averages, vertical bars indicate SEM. Statistically significant between-group differences are marked with an asterisk. (**a**) Distance in meters actively travelled in the AAPA arena as a measure of non-cognitive activity in the task. (**b**) Number of errors. Average number of entries into the punished sector of the arena per session. Transgenic rats made significantly higher amount of spatial errors at the age of 6–7 and 9–10 months, compared to the wild controls. (**c**) Maximal path avoidance. Transgenic subjects scored significantly shorter maximal distance avoided than the wild controls at the age of 6–7 months.

In the analysis of the Active Allothetic Place Avoidance test performance (Figure 1c,d), we initially assessed whether the groups did eventually differ in a non-cognitive parameter of distance travelled on the arena (Figure 2a). The comparison across all subjects revealed significant factors of day, age and sex (*p* < 0.01 in all cases), respectively, and their significant interaction (*p* < 0.01). Subsequent testing at individual age levels returned significant effects in the 4- to 5-month-old control males that revealed a longer path travelled (F (1.14) = 6.54, *p* = 0.023), and in the 6- to 7-month- old female rats showing longer distances travelled in the transgenic group (F (1.13) = 8.52, *p* = 0.012), including a day × group interaction (*p* < 0.05). Animals in the groups of 9 (F (1.33) = 0.63, *p* = 0.432) and 12 months (F (1.36) = 0.05, *p* = 0.818) did not show a significant effect of group or group/sex interaction in distance travelled on the arena. 

**Figure 3:**
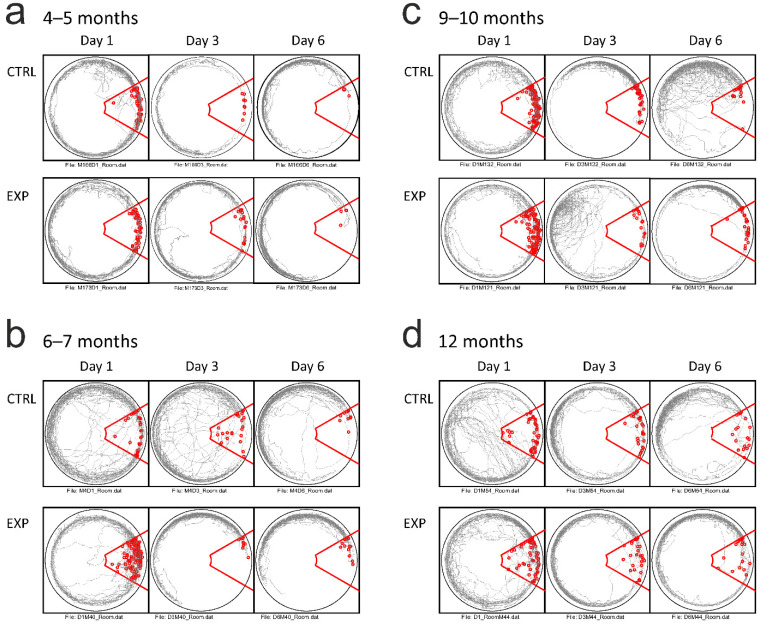
Examples AAPA tracks (grey line) from pairs of experimental and control animals across days 1, 3 and 6 from all age groups. The tracks are depicted in the room coordinate frame. As in Figure 1d, the red line indicates the boundaries of the punished area, with red circles marking the individual footshock’s delivery. Note the evolution of an avoidance behaviour during the course of the training across all age groups: (**a**) 4–5 months, (**b**) 6–7 months, (**c**) 9–10 months and (**d**) 12 months.

In the parameters of spatial memory and navigation, we analysed the maximum path avoided, maximum time avoided, number of spatial errors and the number of shocks received. The comparisons performed across all tested subjects revealed significant effects in factors of age, group and day (all cases *p* < 0.05 in all mentioned parameters, Figure 2b,c and Figure 3 for illustration). In the age-specific comparisons, the transgenic animals showed traces of spatial memory impairment already at the age of 4–5 months: the transgenic subjects made significantly more spatial memory errors normalised for the distance travelled (punished sector entries per meter (F (1.14) = 5.82, *p* = 0.030), and received more shocks (F (1.14) = 10.72, *p* = 0.006)). However, the parameters of maximum time and path avoided did not return significant differences (*p* > 0.225 in both cases). In contrast, the 6-month-old transgenic animals showed markedly impaired task performance, compared to their age-matched controls across all spatial memory parameters reported. They scored a higher number of spatial errors (punished sector entries, F (1.35) = 9.29, *p* = 0.004, confirmed after correction for the distance travelled F (1.35) = 5.34, *p* = 0.027), as well as exhibiting a shorter maximal path avoided (F (1.35) = 11.36, *p* = 0.002) and shorter maximal time avoided (F (1.35) = 8.38, *p* = 0.006, Figure 2). The difference in the number of shocks delivered in the punished sector did not reach the significance level (F (1.35) = 3.62, *p* = 0.065).

At the age level of 9–10 months, the transgenic subjects showed impairment in the number of spatial errors (F (1.35) = 8.07, *p* = 0.007, F (1.35) = 4.62, *p* = 0.039 after correction for individual distance travelled) and maximum time avoidance (F (1.35) = 4.31, *p* = 0.045). They did not significantly differ in the maximum path avoided (F (1.35) = 1.13, *p* = 0.295, Figure 2) and in the number of shocks received (F (1.35) = 3.08, *p* = 0.088). At the age of 12 months, the transgenic and control groups did not significantly differ in any of the reported parameters (all cases *p* > 0.05, Figure 2). We did not observe a significant factor of sex in any of the spatial cognition related parameters within any of the age-specific comparisons (Figures S2 and S3).

Comparisons across all age levels within the control groups showed progressive performance deterioration in all spatial memory measurements. Both 4- and 6-month-old control animals significantly differed from their 9- to 10- and 12-month mates in the number of normalised spatial errors (all *p* < 0.05). In addition, the 6- to 7-month-old subjects were superior in the maximum path and maximum time avoidance than both older control groups (*p* < 0.05 in all four comparisons). The 4- and 6-month-old control groups did not significantly differ from each other (all *p* > 0.05). The age-related differences were less pronounced within the transgenic individuals. In the number of normalised spatial memory errors, the two younger transgenic groups scored better than the 9- to 10-month-old experimental group (*p* < 0.05), while they did not significantly differ from each other and from the 12-month-old experimental subjects (*p* > 0.05). There was no significant difference among the transgenic groups of any age in maximum avoiding path and time (all *p* > 0.05, Figure 2). 

Finally, we assessed the numbers of shocks received per spatial error—a spatial memory-independent component of the performance that rather reflects a procedural form of learning. The analysis revealed that all the groups of animals, independent of age and phenotype, optimised their behaviour, as they significantly decreased their respective scores across the 6-day procedure (*p* < 0.0001). There was a significant group × age interaction (F (3.186) = 15.33, *p* = 0.043). The post-hoc testing found that the 4- to 5-month transgenic rats received significantly more shocks per single spatial memory error than their age-matched controls (*p* < 0.05). The transgenic and control animals from the other age groups did not differ significantly. 

## 4. Discussion

Our results describe the development of spatial memory impairment in the transgenic rat model of Alzheimer’s disease TgF-344 AD. The findings indicate that the impairment is clearly expressed in 6- to 7-month-old animals, while the first signs of the suboptimal spatial behaviour start at the age of 4 to 5 months. 

The question of the development of spatial memory impairment in the TgF-344 AD model has to date been raised by several studies with different results [11,25], probably originating from a difference in the respective testing methods. In the original report by Cohen et al. (2013), the Barnes Maze was used to assess spatial memory performance across groups of 6, 15 and 25 months of age. While at the age of 6 months, the transgenic animals did not differ from wild subjects, the 15- and 25-month-old transgenic groups showed significantly inferior spatial memory than their respective controls. In the more recent study by Berkowitz et al. (2018), the Morris Water Maze procedure was employed and animals at the age of 4–5, 7–8 and 10–11 months were tested. Clear spatial memory impairment was detected in the 10- to 11-month-old transgenic subjects. However, signs of suboptimal behaviour, in the form of a lower proportion of direct paths towards the goal, were apparent already in 7- to 8-month-old transgenic rats. Other studies on TgF-344 AD rats reported the early impairment onset in a variety of other non-spatial behavioural tests: increased anxiety-like behaviour beginning between 4 and 6 months in Pentkowski et al. (2018), hypoactivity in open field and hyposmia beginning at 6 months of age in Saré et al. (2020), and an impairment in the delayed non-match to sample test already at the age of 5 months [33]. 

Because numerous findings indicated that the first cellular and physiological pathologies appeared in TgF-344 AD between 4 and 6 months [11,24,33], we hypothesised that, by using more sensitive behavioural tests, we could better estimate the actual spatial memory impairment onset. The Active Allothetic Place Avoidance enables a selective engagement of the distant landmarks navigation (allocentric), and, at the same time, it requires a suppression of the local and self-motion cues (idiothetic, egocentric orientation). This imposes a qualitatively higher demand on hippocampal memory processing than the commonly used spatial orientation procedures. The AAPA protocol showed the spatial memory was impaired in the TgF-344 AD rats at a younger age than reported previously [11,25]. We observed the deterioration effect across all reported parameters at 6–7 months of age, although the traces of mild deterioration (spatial errors per distance travelled) were detected already in the 4- to 5-month groups. The transgenic animals made more spatial errors and were able to avoid the punished sector for a shorter time or path. This effect was still present in 9- to 10-month-old animals. The oldest animal groups performed equally, irrespective of their phenotype. We interpret the absence of the effect in 12-month-old rats as being the general effect of aging, that also worsened the performance in the control group. Except for the 4- to 5-month-old animals that were only males, we were able to compare the performance across both sexes. Despite rather subtle differences in locomotor activity (higher distance travelled by 6- to 7-month-old transgenic females), we did not find any sex-specific effect. 

In humans, selective allocentric navigation testing has been shown to be effective in the detection of mild cognitive impairment as a consequence of ageing, as well as in early Alzheimer’s patients [16,17,34]. The data suggest that allocentric information processing is weakened with age in AD, and is compensated for by egocentric navigation strategies [16]. Such a proposed shift in navigation approaches might correspond to our results, where the impairment was first detected in the younger transgenic animals and then disappeared after the controls reached 12 months, when their ability to process allocentric information became less effective. 

An effect of sex was observed in the vision test performance. The visual platform version of the Morris Water Maze procedure revealed a progressive deterioration of vision in both sexes, but with different dynamics. While in male subjects we detected a progressive impairment across both transgenic and control groups without any significant difference, in females there was a marked shift in the onset of vision impairment towards the higher age. Whereas at 6–7 and 9–10 months of age, the females did score literally without any impairment, the 12-month-old animals expressed a severe drop in their performance, especially the transgenic ones. This assessment thus showed that F-344 females at younger ages were more resistant to vision deterioration than males. At the higher age level, the onset of sight impairment probably starts as described by Tsai et al. (2014) [35]. In this study, the authors reported a visual impairment in 14- and 19-month-old TgF-344 AD individuals. They showed the first signs of β-amyloid and later plaque formation in the retina, together with the complement activation and inflammatory changes. As pointed out, these effects were detected in animals older than those we used, which might explain their absence in our transgenic groups, except for the female 12-month-old TgF-344 ADs. 

In addition to cognitive decline, Alzheimer’s disease is associated with the presence of neuropsychiatric symptoms, such as anxiety, depression, apathy and behavioural disturbances, which appear in the early stages of the disease [36,37]. We thus set out to supplement the spatial memory testing with the assessment of anxiety-like behaviour in the Elevated Plus Maze, to investigate the possible co-evolution of impairment in the cognitive and non-cognitive domains.

In the anxiety test, the groups were rather statistically indistinguishable, except for the male subjects of 9–10 months of age. Here the transgenic animals showed a higher proportion of time spent in the closed arms of the maze. However, their performance was rather comparable to other age groups, and the difference might possibly have been driven by the drop in scores obtained in the control group. Our results from the Elevated Plus Maze test are thus in partial contrast to the study by Pentkowski et al. (2018). However, they might have arisen from the differences in the maze construction (e.g., the Elevated Plus Maze apparatus in Pentkowski et al. (2018) had transparent arms, that might have increased its sensitivity). In general, the early onset of increased anxiety signs shown in TgF-344 AD rats ([11] and here) corresponds well with the findings in Alzheimer’s patients, reporting anxiety symptoms among the early manifestations of the disease [36]. In addition to the hippocampus and the prefrontal cortex, the amygdala is a region that shows a higher accumulation of amyloid-β and neurofibrillary tangles, including cell loss [38,39] that might point towards the emotional disturbances found both in human subjects and in the animal models.

At the age of 6 months, the TgF-344 AD rats already present a significant Tau pathology, while considerable amyloid plaque formation emerges only in more advanced stages of disease progression [11]. The Tau-mediated network dysfunction thus might be an important driver of the observed cognitive impairment in young TgF-344 AD rats. These findings further emphasise the need for recapitulating a full range of AD-related pathology in translational models of the disease.

## 5. Conclusions

We report a systematic investigation into the development of spatial learning and memory impairment in a rat model of Alzheimer’s disease TgF-344 AD. Using the experimental protocol of split orientation frames with a high sensitivity to mild damage within the hippocampo-entorhinal circuitry, we found that spatial memory impairment was robustly expressed in transgenic animals already at the age of 6–7 months. Moreover, in some navigation parameters, the performance deteriorated even at the age of 4–5 months. Because the impairment was detected at an age with the minimal presence of amyloid in the brain tissue, these findings might contribute considerably to the discussion about the relation between structural and functional damage in Alzheimer’s disease models. 

## Data Availability

Datasets used in this publication are available on request at the corresponding authors.

## Supplementary Materials

The following are available online at www.mdpi.com/article/10.3390/brainsci11101300/s1, Table S1: Visible platform Water Maze task, Figure S1: Visible platform Water Maze task performance across sexes, Figure S2: Active Allothetic Place Avoidance task performance in female groups, Figure S3: Active Allothetic Place Avoidance task performance in male groups.
