# Supplementary material for: Early Spatial Memory Impairment in a Double Transgenic Model of Alzheimer’s Disease TgF-344 AD
Source: Brain Sci. 2021 Sep 30;11(10):1300. doi: 10.3390/brainsci11101300 (PMC8533693; doi:10.3390/brainsci11101300)
Supplement: Supplementary file 1 [file brainsci-11-01300-s001.zip › brainsci-1371510-supplementary.pdf]

age	sex	group	passed	excluded	all	Chi square	p
4–5	M	CTRL	8	0	8	0	1.000
		EXP	8	0	8		
6–7	F	CTRL	8	0	8	0	1.000
		EXP	7	0	7		
	M	CTRL	11	2	13	0	1.000
		EXP	11	2	13		
9–10	F	CTRL	11	1	12	0.529	0.467
		EXP	6	0	6		
	M	CTRL	11	4	15	0.277	0.599
		EXP	9	5	14		
12	F	CTRL	12	6	18	4.620	0.032
		EXP	10	19	29		
	M	CTRL	11	8	19	0.089	0.765
		EXP	9	8	17		

Table S1.
Visible platform water maze task. The table indicates the amounts of animals that passed the test and were further tested in AAPA procedure, rats that did not reach the criterion and were excluded and the total amount of animals that entered the study. The between group comparisons are reported by chi square statistics.

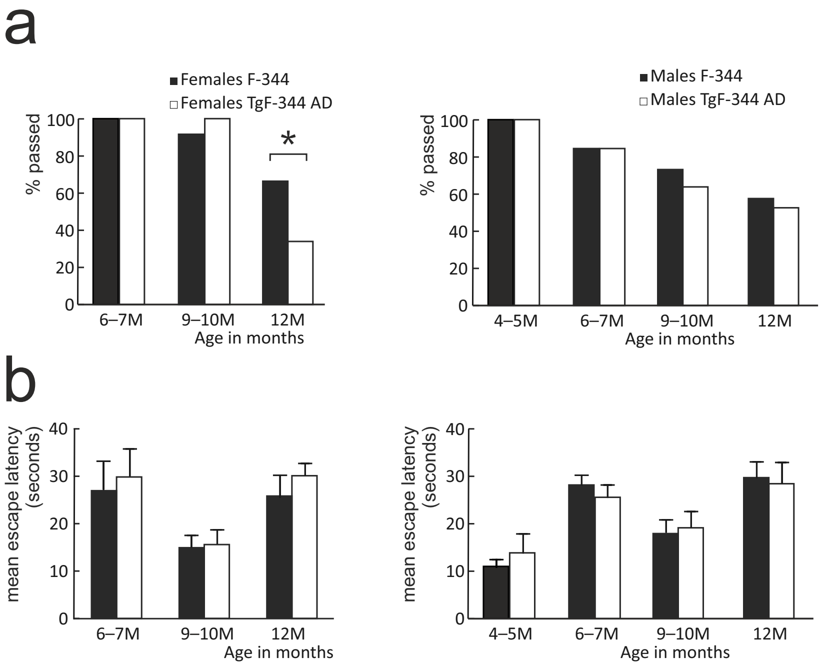

Figure S1.

Visible platform water maze task performance across sexes. (a) The proportion of animals that met the criterion in percents. (b) Average escape times from the second day trials in the animals that passed the test. The vertical bars indicate the SEM. There was no significant difference across the transgenic and wild groups. Females on the left, males on the right.

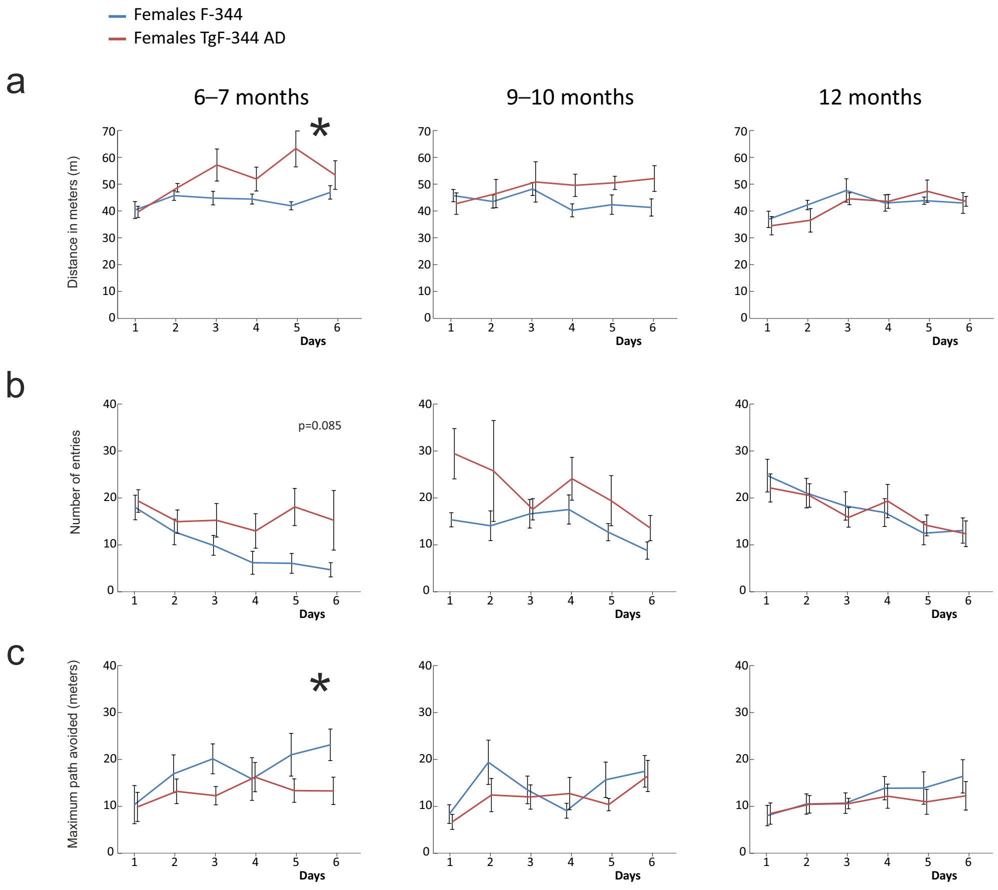

Figure S2.

Active Allothetic Place Avoidance task performance in female groups. Transgenic animals are marked in red, wild type animals in blue. Values are depicted as group averages including SEM. Statistically significant between-group differences are marked with an asterix.

(a) Distance in meters actively traveled in the AAPA arena as a measure of non-cognitive activity in the task. The females from the youngest age group scored significantly longer distance on the arena.

(b) Number of errors. Average number of entries into the punished sector of the arena per session. Female transgenic rats made significantly higher amount of spatial errors at the age of 6–7 months (after normalization by distance walked, not shown) compared to wild controls.

(c) Maximal path avoidance. Transgenic female subjects scored significantly shorter maximal distance avoided than the wild controls at the age of 6–7 months.

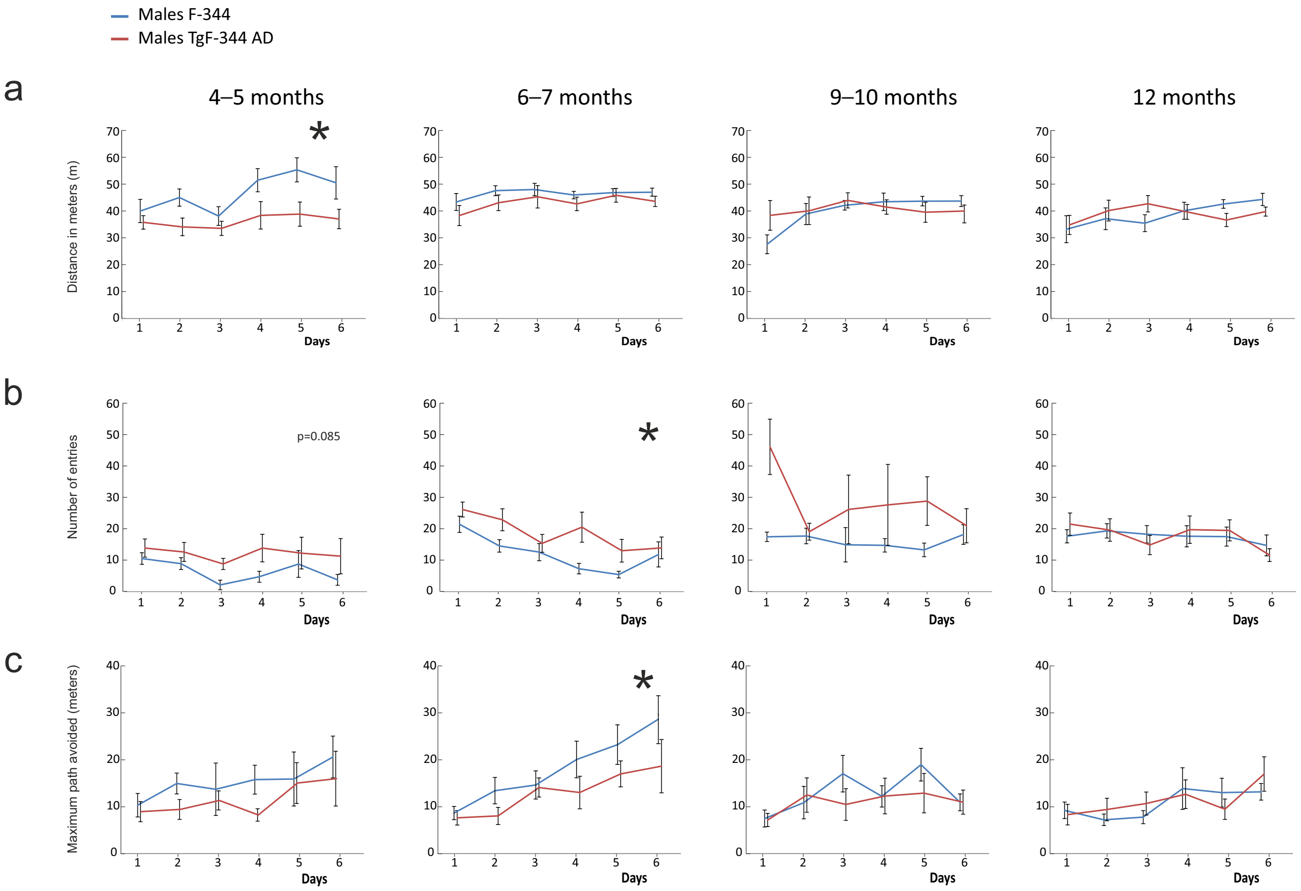

Figure S3.

Active Allothetic Place Avoidance task performance in male groups. Transgenic animals are in red, wild type animals in blue. Values are depicted as group averages including SEM. Statistically significant between-group differences are marked with an asterix.

(a) Distance traveled in the AAPA arena as a measure of non-cognitive activity in the task. The 4–5 months old transgenic male rats scored significantly shorter distance on the arena.

(b) Number of errors. Average number of entries into punished sector of the arena per session. Male transgenic rats made significantly higher amount of spatial errors at the age of 6–7 months compared to wild type controls.

(c) Maximal path avoidance. Male transgenic subjects scored significantly shorter maximal distance avoided than the wild controls at the age of 6–7 months.
